# *In Silico* Identification and Characterization of Drug Targets in *Streptococcus pneumoniae* ATCC 700669 (Serotype 23F) by Subtractive Genomics

**DOI:** 10.1155/2024/5917667

**Published:** 2024-01-20

**Authors:** Tolossa Duguma, Hunduma Dinka

**Affiliations:** ^1^Department of Biotechnology, Wolkite University, Wolkite, Ethiopia; ^2^Department of Applied Biology, Adama Science and Technology University, Adama, Ethiopia

## Abstract

*Streptococcus pneumoniae (S. pneumoniae)* is an important pathogen worldwide that causes pneumococcal infections which are related to high rates of morbidity and mortality especially in young children, older adults, and immune-compromised persons. Antibiotic resistance in *S. pneumoniae* is a serious problem across the world from time to time, resulting in treatment failure and diminished value of older medicines. Therefore, the objective of this study was to identify new putative drug targets against *S. pneumoniae* serotype 23F by using subtractive genomics. By using bioinformatics tools such as NCBI, UniProt KB, PDB, KEGG, DEG, PSORTb, CD hit, DrugBank database, and other softwares, proteins involved in unique metabolic pathways of *S. pneumoniae* serotype 23F were studied. The result indicates that this serotype consists of 97 metabolic pathways of which 74 are common with that of human, and 23 pathways are unique to the serotype 23F. After investigation and analysis of essentiality, nonhomology, subcellular localization, having drug targets, and enzymatic activity, four proteins were prioritized as druggable targets. These druggable proteins include UDP-N-acetylglucosamine 1-carboxyvinyltransferase, UDP-N-acetyl muramate dehydrogenase, D-alanine-D-alanine ligase, and alanine racemase that are found in *S*. *pneumoniae* serotype 23F. All these four proteins are essential, are nonhomologous with human proteins, have drug targets, and are located in cell cytoplasm. Therefore, the authors recommend these proteins to be used for efficient drug design against *S*. *pneumoniae* serotype 23F after experimental validation for essentiality and druggability.

## 1. Introduction

The human pathogen *S. pneumoniae* is one of the leading causes of illness and mortality particularly in children under five years age, adults above 60 years, and immuno-compromised individuals [1]. *S*. *pneumoniae* is encapsulated gram-positive, diplococcal bacteria, an aerotolerant anaerobe, and alpha-haemolytic and is considered the main cause of bacterial pneumonia, otitis media, meningitis, sepsis, and conjunctivitis. The organism is able to translocate from the niche to other host tissues causing a spectrum of diseases referred to as invasive pneumococcal disease. It can also cause osteomyelitis, septic arthritis, acute sinusitis, endocarditis, peritonitis, pericarditis, cellulitis, and brain abscess [2]. It is the leading cause of community-acquired pneumonia and is considered to be a major cause of death of children under five years old worldwide. A report on global antibiotic resistance published by WHO in 2014 indicates that pneumococcus was considered to be one of the nine bacteria of international concern. For instance, in bacterial meningitis, pneumococcus is associated with mortality rates ranging from 16% to 37% [3].

The most important virulence factors in pneumococci include the capsular polysaccharides, adherence factors, invasion genes, heavy-metal transporters, host defence evasion, pneumolysin production, bacteriocin production, quorum sensing, and biofilm formation. The capsule is the most important virulence factor. Over 90 different capsular types of *S. pneumoniae* have been characterized. Some serotypes of the pneumococcus may be carried in the nasopharynx without symptoms, with disease occurring in a small proportion of infected individuals. Other serotypes are rarely identified in the nasopharynx but are associated with invasive disease. The organism may spread locally into the sinuses or middle ear cavity, causing sinusitis or otitis media. It may also affect the lungs to cause pneumonia or cause systemic infections including bacteraemic pneumonia, bacteraemia, and meningitis. Invasive pneumococcal disease refers to a disease in which the bacterium enters a sterile site such as blood, cerebrospinal fluid, pleural fluid, or pericardial fluid. Noninvasive disease includes otitis media, sinusitis, and bronchitis. Invasive pneumococcal disease is a leading cause of morbidity and mortality in both children and adults. According to a literature review, serotypes 1, 4, 5, 7F, 8, 12F, 14, 18C, and 19A are more likely to cause invasive pneumococcal disease [4].

Discovery of penicillin and other antibiotics in the last century revolutionized the treatment of pneumococcal infections due to its sensitivity to penicillin and other beta-lactam antibiotics. Beta-lactam antibacterial agents such as cephalosporins, penicillins, and carbapenems have been used for over four decades in the treatment of *S*. *pneumoniae* infections. These substances bind to high-molecular-weight penicillin-binding proteins, which inhibits the last stages of peptidoglycan production. Beta-lactam nonsusceptible pneumococcal isolates can be treated with macrolides (erythromycin), fluoroquinolones (levofloxacin), lincosamides (clindamycin), and vancomycin [5]. However, since the 1980s, there has been a growth in antibiotic resistance among *S. pneumoniae* which is a serious problem across the world [6]. There is widespread worry about increased levels of antibiotic resistance as well as concerns that the efficacy of antimicrobial therapy may be at risk, resulting in treatment failure. Resistance of pneumococcus against *β*-lactams and macrolides is a major concern worldwide [7]. A higher risk of infections resistant to macrolides has been seen in cases associated with specific pneumococcus vaccine serotypes that include serotypes 14, 6B, 19F, 19A, 9V, and 23F [8].

Primary biochemical pathways of resistance that are used by bacteria to resist the effects of antimicrobial medicines include the enzymatic modification or destruction of the antibiotic, alteration of the antibiotic target site, simulation of the antibiotic target with similar biochemical functions or overproducing the antibiotic target, reduction in antibiotic penetration, and removal of the antibiotic from the cell by efflux pumps [9].

In order to increase the efficacy of antibiotics, mining uncharacterized genes of pathogens to identify potential targets for entirely new classes of antibiotics becomes an alternative practice [10]. Khan et al. [11] identified 47 potent drug targets in S. pneumonia serotype 14 by using metabolic pathways analysis and subtractive genomics in which they prioritized two proteins, i.e., 4-oxalocrotonate tautomerase and sensor histidine kinase. Sheoran et al. [12] also identified 28 essential nonhomologous proteins of which they screened 5 druggable proteins and prioritized 3 cytoplasmic proteins in *S*. *pneumoniae* D39 strain by using subtractive genomics. Since there are over 90 serotypes of *S*. *pneumoniae* and only a few of them have been studied so far, it is important to conduct research on these serotypes regarding drug targets and mechanism of drug resistance to fill the gap. Without knowing the biochemical function of a protein, it is difficult to validate its potential for drug targeting. Therefore functional characterization of bacterial proteins of unknown function must be enhanced. The most attractive targets for new classes of antibiotics are proteins involved in previously untargeted essential biochemical pathways conserved among pathogens and absent in humans. Understanding the function of these proteins is a prerequisite for the validation of targets for the development of antibacterial drugs with novel mechanisms of action [13]. Therefore, the study was aimed at identifying new putative drug targets against *S. pneumoniae* serotype 23F by using several bioinformatics tools and subtractive genomics to recognize the possible putative drug targets.

## 2. Materials and Methods

### 2.1. Gene and Protein Sequence Retrieval from Databases

The complete genome of *Streptococcus pneumoniae* ATCC 700669 serotype 23F was retrieved from the NCBI database using GenBank accession number FM211187.1. NCBI can be freely accessible from the website (http://www.ncbi.nlm.nih.gov). Protein sequences were also retrieved from the UniProt database (https://www.uniprot.org/). *S. pneumoniae* genome database annotation was used to find proteins having human homologs, and comparative BLAST was performed using NCBI's BLAST.

### 2.2. Analysis of Metabolic Pathways in *S. pneumoniae*

The analysis of metabolic pathways was used to find the unique pathways which are not present in the host by using the KEGG (Kyoto Encyclopedia of Genes and Genomes) database (http://www.kegg.jp). KEGG ID number T00843 was used for searching metabolic pathways of *S. pneumoniae*, while KEGG ID number T01001 was used for searching human metabolic pathways by selecting *Homo sapiens* name as an organism. After *s*earching saved pathways in KEGG for both the host and the pathogen, pathways of the pathogen were manually compared with human pathways to find the pathogen's unique pathways so that the pathways of the host will not get disturbed due to drugs [14].

### 2.3. Elimination of Homologous Protein Sequences of Pathogens

For the elimination of homologous proteins from nonparalogous datasets of pathogen, BLASTp was used against Reference Sequence *Homo sapiens* by selecting the *E* − value < 10^−3^ that is basically the cutoff expectation [15]. BLASTp showed two different results. The first one was those protein sequences which are similar to the host sequences that show significant similarity, and the second one was those sequences which are nonhomologous sequences. Nonhomologous sequences were used for further analysis while the homologous sequences were eliminated.

### 2.4. Determination of Essential Genes of Pathogen

The information about essential genes was collected from databases, i.e., DEG, which is freely accessible from the website (http://origin.tubic.org/deg). DEG is developed for crucial genes and proteins, which are extracted from different reports, scientific papers, and experimental procedures [16]. The DEG database was utilized for the determination of nonhost crucial genes by selecting the *E* − value < 10^−5^ that is basically the cutoff expectation during BLAST.

### 2.5. Finding Nonparalogous Sequences

CD-HIT cluster database for high identity with a sequence identity criterion of 80% was utilized to identify paralogous or duplicate protein successions. Duplicate sequences were eliminated from *S. pneumoniae* serotype 23F entire proteins, leaving only nonparalogous sequences.

### 2.6. Subcellular Localization Prediction

Unique proteins were analyzed through the KEGG pathway analysis and used for the prediction of localization by an online localization tool called PSORTb3.0. PSORTb3.0 was used to find subcellular localization of unidentified proteins, and it can be easily accessible from the website (http://www.psortb.org).

### 2.7. Druggability Target Analysis

**Figure 1:**
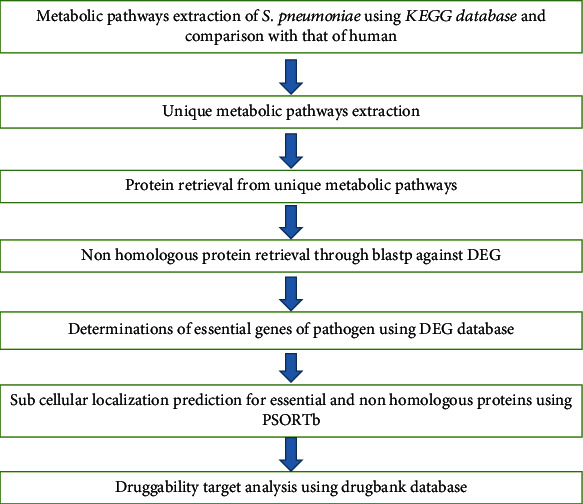
Flowchart for methodology (12) with some modification.

For the identification of druggable targets of pathogen proteins, the DrugBank database was used. It is freely accessible from the website (https://go.drugbank.com). The proteins that are only belonging to unique pathway or unique proteins were subjected to DrugBank to find their targets one by one to check the druggability of those proteins. The screening of all essential, nonhomologous that are involved in unique metabolic pathways were evaluated by BLASTp comparing against the database of DrugBank which consists of the number of protein targets which serve as a novel drug target against the invasive disease caused by the *S. pneumoniae* serotype 23F [17]. Procedures followed were briefly explained by the flowchart (Figure 1).

## 3. Results and Discussions

### 3.1. Gene and Protein Sequence Retrieval from Databases

The whole genome of *Streptococcus pneumoniae* ATCC 700669 serotype 23F retrieved from the NCBI database indicates that its complete genome consists of 2.2 Mb of DNA with a GC content of 39% and a total of 2,191 genes which codes for proteins and other regulatory elements. UniProt KB database search results also indicate that *S. pneumoniae* ATCC 700669 serotype 23F consists of 1,992 proteins.

### 3.2. Analysis of Metabolic Pathways in *S. pneumoniae*

Since metabolic pathways involve many different types of essential proteins which participate in different cellular functions, it is very important to identify common metabolic pathways and unique metabolic pathways found in a given microorganism. Unique pathways are classified as those pathways that are present only in the pathogen, while common metabolic pathways are those pathways that are present in both the pathogen and the host [18]. Accordingly, the analysis of metabolic pathways using the KEGG database indicates that there is a total of 97 metabolic pathways in *S. pneumoniae* serotype 23F among which 74 metabolic pathways were found to be common for both host (human) and pathogen (*S. pneumoniae serotype* 23F). The rest 23 metabolic pathways were unique to *S. pneumoniae serotype* 23F. Since the objective of the current study was to identify essential and nonhomologous proteins found in unique metabolic pathways of *S. pneumoniae serotype* 23F, proteins of common metabolic pathways were discarded while all the proteins found in this unique pathways were retrieved from UniProt and further studied for their essentiality, homology, and druggability.

The unique metabolic pathways found only in *S. pneumoniae* serotype 23F involve pathways that are important for pathogen in drug resistance mechanisms such as beta-lactam resistance, vancomycin resistance, and cationic antimicrobial peptide (CAMP) resistance. The unique metabolic pathways found in the pathogen involve 488 proteins (Table 1). Among these proteins, many of them are homologous with that of human proteins, and some of them are nonessential for the survival of *S. pneumoniae*. By conducting further analysis, homologous proteins and nonessential proteins were discarded, and only nonhomologous and essential proteins were screened and further analyzed for drug targets.

### 3.3. Identification of Nonhomologous and Nonparalogous Proteins

In order to identify nonhomologous proteins, all the 488 protein sequences retrieved from the unique metabolic pathways were subjected to BLASTp against Homo sapiens using the KEGG database to identify nonhomologous proteins. Out of 488 proteins involved in unique metabolism pathways of *S. pneumoniae* serotype 23F, only 103 proteins were identified as both nonhomologous with human proteins and nonparalogous. Nonhomologous proteins are proteins of the pathogen that have no similar sequences in human based on amino acid sequence similarity criteria. The essential protein that has a druggable target must also be nonhomologous with that of human protein so that the human proteins cannot be affected by drugs that affect pathogen proteins.

### 3.4. Determination of Essential Genes of Pathogen and Subcellular Protein Prediction

The information about essential genes was collected from DEG databases. The DEG database was utilized for the determination of nonhost crucial genes that are present in *S. pneumoniae* serotype 23F. Accordingly, 244 genes that code for essential proteins were identified. All the above 103 proteins identified as nonhomologous and nonparalogous proteins found in unique pathways of *S. pneumoniae* ATCC700669 (serotype 23F) were searched for their essentiality by using BLASTp against DEG and *E*-value 10^−5^. Finally, 21 proteins were identified as nonhomologous, nonparalogous, and essential proteins of *S. pneumoniae* ATCC700669 serotype 23F (Table 2).

Since these proteins are essential for the survival of the bacteria, all these 21 proteins can be used as drug targets. However, by analyzing for druggability and cellular location of these proteins, it was further prioritized for drug targets. Among these proteins that were identified as essential, nonhomologous, and nonparalogous proteins in unique pathways of *S. pneumoniae* 23F, four of them were found to be cytoplasmic membrane proteins while the rest 17 proteins were found to be cytoplasmic proteins (Table 2). Cytoplasmic proteins are more suitable for drug target as it is difficult to purify the proteins that are present in the membrane of the cell [19]. Moreover, surface proteins have direct contact, so these protein targets have the ability to obtain an immune response when defined to the host. So it is preferable for the vaccine target rather than using it as a drug target.

### 3.5. Druggability Target Analysis

The screening of all essential and nonhomologous proteins that are involved in unique metabolic pathways was evaluated by BLASTp comparing against the database of DrugBank which consists of a number of protein targets that serve as a novel drug target against the invasive disease that caused by the pathogen. Among these 21 proteins that are essential and nonhomologous with human proteins, only five proteins showed druggable targets for FDA-approved drugs. However, among the shortlisted five proteins (Table 3) that have drug targets, one is found to be cytoplasmic membrane protein. Therefore, it was discarded, and four cytoplasmic proteins that have drug targets were prioritized. Based on different criteria such as subcellular localization, having druggable targets for drugs and enzymatic activity of specific essential and nonhomologous proteins, the shortlisted proteins are UDP-N-acetylglucosamine 1-carboxyvinyltransferase, UDP-N-acetyl muramate dehydrogenase, D-alanine-D-alanine ligase, and alanine racemase as druggable proteins found in *S. pneumoniae* serotype 23F.

The study conducted by Khan et al. identified 47 proteins which are essential, nonhomologous, and those having drug target against *S. pneumoniae* serotype 14 [11]. However, we have identified only 5 proteins which are essential, nonhomologous, and those that have drug target against *S. pneumoniae* serotype 23F. This variation may be due to serotype difference. But [12] identified 5 proteins which are essential, nonhomologous, and that have drug target in *S. pneumoniae D39* strain which is inline with our work.

#### 3.5.1. UDP-N-Acetylglucosamine 1-Carboxyvinyltransferase (murZ)

This protein has druggable targets for fosfomycin (DrugBank Accession Number DB00828) which is approved drug by FDA [20]. Despite being FDA-approved for urinary tract infections, fosfomycin has a broad spectrum of activity and is active against both gram-positive and gram-negative bacteria. The protein has UDP-N-acetylglucosamine 1-carboxyvinyltransferase activity and is important in bacterial cell wall formation by adding enolpyruvyl to UDP-N-acetylglucosamine. The gene that codes for the protein is known as *murZ*. The molecular weight of the protein is 44817.24 Da. The DrugBank database BLASTp shows that the alignment with fosfomycin resulted in *E*-value: 3.53435e-^91^ that is statistically significant, and it indicates that the protein has a druggable target for this drug.

The protein has also druggable targets for experimental drugs such as (S)-2-{methyl-[2-(naphthalene-2-sulfonylamino)-5-(naphthalene-2-sulfonyloxy)-benzoyl]-amino} succinicacid, aminomethylcyclohexane, cyclohexylammonium ion, 3′-1-carboxy-1-phosphonooxy-ethoxy-uridine-diphosphate-N-acetylglucosamine, and 8-anilinonaphthalene-1-sulfonic acid. The protein participates in pathways such as amino sugar and nucleotide sugar metabolism, peptidoglycan biosynthesis, metabolic pathways, and biosynthesis of nucleotide sugars. Even though *MurZ* alone is not enough to cause penicillin resistance, it is important in the acquisition of the highest level of resistance to both penicillin and cephalosporin antibiotics [21]. Therefore, targeting such protein in *S. pneumoniae* is very important in combating antibiotic resistance.

*S. pneumoniae* harbour two genes (*murA* and *murZ*) encoding UDP-*N*-acetylglucosamine enolpyruvyl transferase activity for catalyzing the first committed step of peptidoglycan biosynthesis. The *S. pneumoniae* MurA and MurZ genes encode isozyme proteins that exhibit 45.8% amino acid sequence identity and are similar in length 427 residues (MurA) and 419 residues (MurZ). The reaction between UDP-*N*-acetylglucosamine (UDPGlcNAc) and phosphoenolpyruvate (PEP) catalyzed by UDP-GlcNAc enolpyruvyl transferase enzymes is the first committed step in the biosynthesis of peptidoglycan by bacteria [22]. According to [23], the independent inactivation of either *murA* or *murZ* is not lethal in *S. pneumoniae*, indicating that either isozyme can sustain viability. Therefore, it is better to target both *murA* and *murZ* genes for therapeutic target rather than targeting either of them. However, conducting in vitro experiment is necessary to identify which gene is more essential for a particular strain or serotype by deleting either of the genes from the organism and evaluating its survival.

#### 3.5.2. UDP-N-Acetyl Enol Pyruvoyl Glucosamine Reductase (murB)

UDP-N-acetyl enol pyruvoyl glucosamine reductase protein has druggable targets for flavin adenine dinucleotide (FAD). FAD (DrugBank Accession Number DB03147) is an approved drug by the FDA to be used by human, and it is found under the list of FDA-approved drugs. The gene that codes for the protein is known as *murB*. The DrugBank database BLASTp shows that the alignment with flavin adenine dinucleotide drug resulted in *E*-value: 2.6683e-91, which is statistically significant and indicates the druggability of the protein with this drug. UDP-N-acetyl enol pyruvoyl glucosamine reductase has also druggable targets for some experimental drugs such as (5Z)-3-(4-CHLOROPHENYL)-4-HYDROXY-5-(1-NAPHTHYLMETHYLENE)FURAN2(5H)- ONE. However, the side effects of such experimental drug must be studied by researchers and approved by the FDA prior to use as an antibacterial drug. The molecular weight of UDP-N-acetyl enol pyruvoyl glucosamine reductase is 23448.95 Da. The protein participates in pathways such as amino sugar and nucleotide sugar metabolism, peptidoglycan biosynthesis, and biosynthesis of nucleotide sugar pathways.

UDP-N acetyl enol pyruvoyl glucosamine reductase is encoded by the murB gene and involved in the catalysis of the final steps of the UDP-N-acetylmuramic acid formation [24]. This reaction is an NADPH-dependent reduction of enolpyruvyl-UDP-N-acetylglucosamine, releasing UDP-*N*-acetylmuramic acid as a product, to which three amino acids will subsequently be added sequentially by other enzymes in the pathway [25]. MurB is a flavoprotein and belongs to the superfamily category of FAD binding protein with a feature of flavin adenine dinucleotide binding fold [26]. This result is also inline with the current study that indicates that UDP-N-acetyl enol pyruvoyl glucosamine reductase protein has druggable targets for flavin adenine dinucleotide.

#### 3.5.3. D-Alanine--D-Alanine Ligase (ddl)

This protein has druggable targets for cycloserine **(**DrugBank Accession Number DB00260), a drug that was approved by the FDA. The gene that codes for the protein is known as *ddl*. The DrugBank database BLASTp shows that the alignment with the cycloserine drug shows an *E*-value: 1.25789e-77 which is statistically significant in showing drug targets. The molecular weight of the protein is 39315.435 Da. D-alanine--D-alanine ligase A has a function such as cell wall formation and participates in D-amino acid metabolism, peptidoglycan biosynthesis, metabolic pathways, and vancomycin resistance pathways.

D-alanine: D-alanine ligase catalyzes the ATP-driven ligation of two D-alanine molecules to form the D-alanyl:D-alanine dipeptide. This molecule is a key building block in peptidoglycan biosynthesis, making ddl an attractive target for drug development. The D-alanine racemase (alr) and D-alanine-D-alanine ligase (ddl) enzymes catalyze sequential steps in the D-alanine pathway of peptidoglycan biosynthesis [27]. Both enzymes are inhibited by D-cycloserine in a concentration-dependent manner [28]. Their work is inline with the current study in which we have investigated D-alanine-D-alanine ligase that has a druggable target for FDA-approved drug, cycloserine. The protein has also drug targets for experimental drug called 3-CHLORO-2,2-DIMETHYL-N-[4-(TRIFLUOROMETHYL)PHENYL]PROPANAMIDE.

#### 3.5.4. Alanine Racemase (*Alr*)

The protein has a druggable target for propanoic acid (DrugBank Accession Number DB03766), which is approved by the FDA. The BLASTp of alanine racemase protein and propanoic acid drug results in an *E*-value; 2.17828e^−92^, which is statistically significant and shows drug targets of the protein for this particular drug. Alanine racemase has also druggable targets for many experimental drugs such as N-(5′-phosphopyridoxyl)-D-alanine, pyridoxamine-5′-phosphate, PMP-hydroxyisoxazole, pyridoxamine-5-phosphate-hydroxyisoxazole, {1-[(3-hydroxy-methyl-5-phosphonooxy-methyl-pyridin-4-Ylmethyl)-amino]-ethyl}-phosphonic acid, D-[3-hydroxy-2-methyl-5-phosphonooxymethyl-pyridin-4-ylmethyl]-N,O-cycloserylamide, propanoic acid, and lysine Nz-carboxylic acid. The protein function includes pyridoxal phosphate binding and catalyzes the interconversion of L-alanine and D-alanine. The gene that encodes the protein is known as *alr*, and the molecular weight of the protein is 41001.515 Da. The protein also participates in D-amino acid metabolism, peptidoglycan biosynthesis, metabolic pathways, and vancomycin resistance pathways.

**Figure 2:**
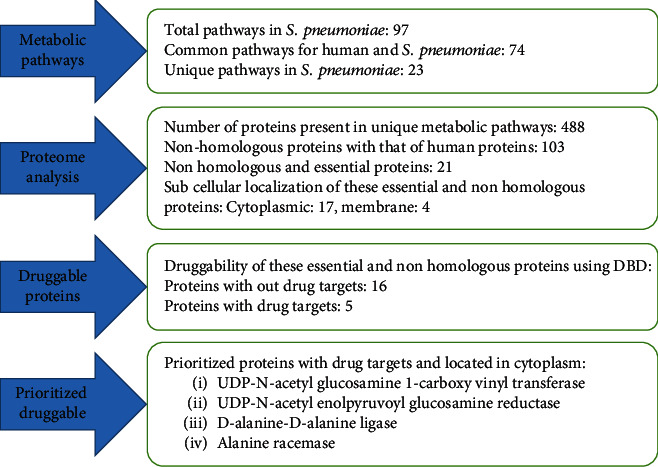
Summary of results.

The gene *alr* encodes the constitutively expressed biosynthetic enzyme alanine racemase that provides D-alanine necessary for peptidoglycan synthesis which is very crucial for bacterial cell-wall biosynthesis. It is an attractive drug target because it is essential for bacterial survival, and it is absent in humans [29]. Alanine racemase is an ubiquitous enzyme among bacteria and provides the essential cell wall precursor, D-alanine [30]. Since it is absent in humans, this enzyme is an attractive target for the development of drugs against *S. pneumoniae* that is inline with the current study. The results obtained were briefly summarized in the following (Figure 2).

## 4. Conclusion and Recommendations

Complete genome of *S. pneumoniae* ATCC 700669 serotype 23F retrieved from NCBI indicates that its genome consists of 2,191 genes of which 1992 codes for proteins. Among these protein-coding genes, 244 gene codes for essential proteins which are important for bacterial survival. The metabolic pathway analysis also indicates that the strain has 97 pathways, of which it shares 74 metabolic pathways with human, and the rest 23 are unique metabolic pathways for the *S. pneumoniae* serotype 23F. In these unique metabolic pathways of the pathogen, 488 proteins are involved, and among them, 103 proteins are nonhomologous with human proteins of which 21 proteins are essential for the *S. pneumoniae*. Since these short-listed 21 essential proteins are also nonhomologous with that of human proteins, they can be used as drug targets, and the host proteins cannot be affected by drugs that target these proteins. Among these essential and nonhomologous proteins, only five of them have druggable targets, but based on different criteria such as subcellular localization, having druggable targets for FDA-approved drugs and enzymatic activity, only four of them were selected and prioritized as druggable proteins. These proteins include UDP-N-acetylglucosamine 1-carboxyvinyltransferase, UDP-N-acetyl muramate dehydrogenase, D-alanine-D-alanine ligase, and alanine racemase.

Even though penicillin-binding protein 2X has a druggable target for FDA-approved drug like cloxacillin and experimental drug 6-o-capryloylsucrose, the authors did not recommend this protein due to its subcellular localization (found in cytoplasmic membrane). Since there is no experimental validation of the targets reported, the authors suggest that experiments should be conducted to validate the essentiality and druggability of these identified and proposed targets. Thus, after experimental validation of the abovementioned drug targets, we recommend them for drug discovery for *S. pneumoniae* serotype 23F.

## Figures and Tables

**Table 1:** Unique pathway for *S. pneumoniae* ATCC 700669 (serotype 23F) (http://www.kegg.jp).

S/N	Pathway IDs	Metabolic pathways	Proteins in the pathway
1.	sne00261	Monobactam biosynthesis	4
2.	sne00300	Lysine biosynthesis	12
3.	sne00332	Carbapenem biosynthesis	2
4.	sne00362	Benzoate degradation	2
5.	sne00460	Cyanoamino acid metabolism	3
6.	sne00521	Streptomycin biosynthesis	7
7.	sne00550	Peptidoglycan biosynthesis	25
8.	sne00622	Xylene degradation	2
9.	sne00625	Chloroalkane and chloroalkene degradation	3
10.	sne00626	Naphthalene degradation	3
11.	sne00660	C5-branched dibasic acid metabolism	5
12.	sne00680	Methane metabolism	8
13.	sne01110	Biosynthesis of secondary metabolites	150
14.	sne01120	Microbial metabolism in diverse environments	82
15.	sne01501	Beta-lactam resistance	7
16.	sne01502	Vancomycin resistance	6
17.	sne01503	Cationic antimicrobial peptide (CAMP) resistance	6
18.	sne02020	Two-component system	39
19.	sne02024	Quorum sensing	62
20.	sne02040	Flagellar assembly	2
21.	sne02060	Phosphotransferase system (PTS)	44
22.	sne03070	Bacterial secretion system	13
23.	sne03250	Viral life cycle-HIV-1	1

**Table 2:** Essential, nonhomologous, and nonparalogous proteins in unique pathways of *S. pneumoniae* 23F (https://www.uniprot.org).

S/N	Gene name	Protein name	UniProt KB ID	Subcellular location	Functions of the proteins
1	asd	L-aspartate-semialdehyde dehydrogenase	B8ZPG6	Cytoplasmic	Isoleucine, lysine, and threonine biosynthetic process, “de novo” L-methionine biosynthetic process
2	pyrH	Aspartate kinase	B8ZLI0	Cytoplasmic	Phosphoribosylaminoimidazolecarboxamide formyltransferase activity, de novo inosine monophosphate biosynthetic process
3	dapB	4-hydroxy-tetra hydrodipicolinate reductase	B8ZLL9	Cytoplasmic	Catalyzes the conversion of 4-hydroxy-tetra hydrodipicolinate to tetra hydrodipicolinate Lysine biosynthesis process
4	MurF	UDP-N-acetylmuramoyl-tripeptide-D-alanyl-D-alanine ligase	B8ZM65	Cytoplasmic	ATP binding, cell cycle, peptide glycan biosynthetic process
5	murZ	UDP-N-acetylglucosamine 1-carboxyvinyltransferase	B8ZPT0	Cytoplasmic	Catalysis of the reaction: phosphoenolpyruvate + UDP-N-acetyl-alpha-D-glucosamine = phosphate + UDP-N-acetyl-3-O-(1-carboxy vinyl)-D-glucosamine
6	murB	UDP-N-acetylmuramate dehydrogenase	B8ZKN7	Cytoplasmic	Peptidoglycan biosynthetic process (uridine diphospho-N-acetyl glucose amine enolpyruvate reductase activity)
7	MurC	UDP-N-acetylmuramate--alanine ligase	B8ZLC2	Cytoplasmic	Cell wall formation; UDP-N-acetylmuramate-L-alanine ligase activity, peptidoglycan biosynthesis
8	MurD	UDP-N-acetylmuramoyl alanine-D-glutamate ligase	B8ZMZ6	Cytoplasmic	Cell wall formation, catalyzes the addition of glutamate to the nucleotide precursor UDP-N-acetyl muramoyl-L-alanine, peptidoglycan biosynthesis
9	ddl	D-alanine-D-alanine ligase	B8ZM66	Cytoplasmic	Cell wall formation, D-alanine-D-alanine ligase activity, ATP binding, cell wall organization, peptidoglycan biosynthesis, regulation of cell shape
10	uppP	Undecaprenyl-diphosphatase	B8ZLV4	Cytoplasmic	Confers resistance to bacitracin, response to antibiotic, and involved in peptidoglycan biosynthetic process
11	pbpX	Penicillin-binding protein 2X	Q4TUL5	Cytoplasmic membrane	Binding to penicillin, an antibiotic that contains the condensed beta-lactam thiazolidine ring system.
12	murE	UDP-N-acetyl muramoyl-L-alanyl-D glutamate-L-lysine ligase	B8ZLD0	Cytoplasmic	Cell division, cell cycle, ATP binding activity, cell wall organization
13	SP_1589	Lipid II isoglutaminyl synthase (glutamine-hydrolysing)	B8ZM05	Cytoplasmic	Carbon-nitrogen ligase activity on lipid II, peptidoglycan biosynthetic process, cell wall organization
14	MurM	Serine/alanine adding enzyme	B8ZMK2	Cytoplasmic	Aminoacyltransferase activity, cell wall macromolecule biosynthetic process
15	Fba	Fructose-bisphosphate aldolase, class II	B8ZMD4	Cytoplasmic	Zinc ion binding, fructose 1,6-bisphosphate metabolic process, glycolytic process
16	rpoD	RNA polymerase primary sigma factor	B8ZPS2	Cytoplasmic	DNA binding, sigma factor activity, DNA-templated transcription initiation
17	secG	Preprotein translocase subunit SecG	B8ZP70	Cytoplasmic membrane	Involved in protein export. Participates in an early event of protein translocation, protein secretion, protein transporting ATPase activity
18	YesM	Two-component system, sensor histidine kinase YesM	B8ZKC2	Cytoplasmic membrane	Protein-transporting ATPase activity, protein transport by the Sec complex
19	PhoP	Two-component system, OmpR family, alkaline phosphatase synthesis response regulator	B8ZPK5	Cytoplasmic	Regulation of DNA-templated transcription, transcription cis-regulatory region binding, phosphorelay response regulator activity
20	alr	Alanine racemase	B8ZMG1	Cytoplasmic	Catalyzes the interconversion of L-alanine and D-alanine. Pyridoxal phosphate binding, D-alanine biosynthetic process
21	murG	UDP-N-acetylglucosamine-N-acetyl muramyl pyro phosphoryl-undecaprenol N-acetylglucosamine transferase	B8ZMZ7	Cytoplasmic membrane	Cell wall formation, carbohydrate metabolic process

**Table 3:** Nonhomologous and essential proteins that have druggable targets for drugs (http://origin.tubic.org/deg).

Gene name	DEG ID	Protein name	Protein ID	Subcellular location
murZ	DEG10070059	UDP-N-acetylglucosamine 1-carboxyvinyltransferase	SPN23F10020	Cytoplasmic
murB	DEG10070176	UDP-N-acetyl enol pyruvoyl glucosamine reductase	SPN23F13540	Cytoplasmic
ddl	DEG10070207	D-alanine-D-alanine ligase	SPN23F16720	Cytoplasmic
pbpX	DEG10070129	Penicillin-binding protein 2X	SPN23F03080	Cytoplasmic membrane
alr	DEG10070209	Alanine racemase	SPN23F16970	Cytoplasmic

## Data Availability

Research data can be presented upon a reasonable request.

## Supplementary Materials

Supplementary Materials Supplementary Table was attached at the end section of this manuscript that indicates a list of nonhomologous and nonparalogous proteins in *S. pneumoniae* 23F.
