# Supplementary material for: In Silico Identification and Characterization of Drug Targets in Streptococcus pneumoniae ATCC 700669 (Serotype 23F) by Subtractive Genomics
Source: Biomed Res Int. 2024 Jan 20;2024:5917667. doi: 10.1155/2024/5917667 (PMC10821801; doi:10.1155/2024/5917667)
Supplement: Supplementary Materials — Supplementary Table was attached at the end section of this manuscript that indicates a list of nonhomologous and nonparalogous proteins in S. pneumoniae 23F. [file 5917667.f1.doc]

1. **Supplementary file**

[Table I. Non homologous and non paralogous proteins in *S. pneumoniae* 23F (www.kegg.jp,](http://cd-hit.org/)  http://cd-hit.org).

| **S/N** | **Protein class** | **Protein name** | **Protein ID** |
| --- | --- | --- | --- |
|  | Aspartate-semialdehyde dehydrogenase | Aspartate-semialdehyde dehydrogenase | SPN23F09380 |
|  | Putative dihydrodipicolinate reductase | 4-hydroxy-tetrahydrodipicolinate reductase | SPN23F15220 |
|  | Putative aspartokinase | Aspartokinase | SPN23F03890 |
|  | Homoserine dehydrogenase | Homoserine dehydrogenase | SPN23F13270 |
|  | Putative transferase | 2,3,4,5-tetrahydropyridine-2,6-dicarboxylate n-succinyl transferase | SPN23F21210 |
|  | Putative peptidase | N-acetyldiaminopimelate deacetylase | SPN23F21200 |
|  | Saccharopine dehydrogenase family protein | Saccharopine dehydrogenase | SPN23F08430 |
|  | Putative UDP-N-acetylmuramoyl-tripeptide--D-alanyl-D-alanine ligase | UDP-N-acetylmuramoyl-tripeptide--D-alanyl-D-alanine ligase | SPN23F16710 |
|  | Putative 4-oxalocrotonate tautomerase | 4-oxalocrotonate tautomerase | SPN23F09410 |
|  | Aspartate--ammonia ligase | Aspartate--ammonia ligase | SPN23F19910 |
|  | Dtdp-4-keto-6-deoxyglucose-3,5-epimerase rmlc | Dtdp-4-dehydrorhamnose 3,5-epimerase | SPN23F03340 |
|  | UDP-N-acetylglucosamine 1-carboxyvinyltransferase 2 | UDP-N-acetylglucosamine 1-carboxyvinyltransferase | SPN23F10020 |
|  | UDP-N-acetylenol pyruvoylglucosamine reductase | UDP-N-acetylmuramate dehydrogenase | SPN23F13540 |
|  | UDP-N-acetylmuramate--alanine ligase | UDP-N-acetylmuramate--alanine ligase | SPN23F14850 |
|  | UDP-N-acetylmuramoylalanine--D-glutamate ligase | UDP-N-acetylmuramoylalanine--D-glutamate ligase | SPN23F06220 |
|  | D-alanine--D-alanine ligase | D-alanine-D-alanine ligase | SPN23F16720 |
|  | Diacylglycerol kinase | Undecaprenol kinase | **SPN23F08930** |
|  | Undecaprenol kinase (bacitracin resistance protein | Undecaprenyl-diphosphatase | **SPN23F04300** |
|  | Putative penicillin-binding protein 1A | Penicillin-binding protein 1A | **SPN23F03410** |
|  | Penicillin-binding protein 2a | Penicillin-binding protein 2a | **SPN23F20310** |
|  | Penicillin-binding protein 1b | Penicillin-binding protein 1b | **SPN23F21230** |
|  | Penicillin-binding protein 2b | Penicillin-binding protein 2b | **SPN23F16740** |
|  | Putative penicillin binding protein 2x | Penicillin-binding protein 2X | **SPN23F03080** |
|  | Putative D-alanyl-D-alanine carboxypeptidase | Serine-type D-Ala-D-Ala carboxypeptidase (penicillin-binding protein) | **SPN23F07950** |
|  | putative aminotransferase | lipid II isoglutaminyl synthase (glutamine-hydrolysing) | **SPN23F16050** |
|  | UDP-N-acetylmuramoylalanyl-D-glutamate--2,6-dia minopimelate ligase | UDP-N-acetylmuramoyl-L-alanyl-D-glutamate-L-lysine ligase | **SPN23F14930** |
|  | Mur ligase family protein | Lipid II isoglutaminyl synthase (glutamine-hydrolysing) | **SPN23F16040** |
|  | Putative aminotransferase | Lipid ii isoglutaminyl synthase (glutamine-hydrolysing) | **SPN23F16050** |
|  | Putative peptidoglycan pentaglycine interpeptide biosynthesis protein | Serine/alanine adding enzyme | **SPN23F05540** |
|  | Putative peptidoglycan branched peptide synthesis protein | Alanine adding enzyme | **SPN23F05550** |
|  | Acetolactate synthase small subunit | Acetolactate synthase i/iii small subunit | **SPN23F04180** |
|  | Alpha-acetolactate decarboxylase | Acetolactate decarboxylase | **SPN23F13560** |
|  | Putative phosphor enolpyruvate carboxylase | Phosphoenolpyruvate carboxylase | **SPN23F09890** |
|  | Fructose-bisphosphate aldolase | Fructose-bisphosphate aldolase, class ii | **SPN23F05450** |
|  | Acetate kinase | Acetate kinase | **SPN23F20660** |
|  | Putative phosphate acetyltransferase | Phosphate acetyltransferase | **SPN23F10200** |
|  | Extracellular solute-binding protein | L-cystine transport system substrate-binding protein | **SPN23F01570** |
|  | RNA polymerase sigma factor rpod | RNA polymerase primary sigma factor | **SPN23F09940** |
|  | Putative conjugal transfer protein trag | Type IV secretion system protein vird4 | **SPN23F12890** |
|  | Preprotein translocase seca subunit-like protein | Preprotein translocase subunit seca | **SPN23F17680** |
|  | Putative preprotein translocase sece subunit | Preprotein translocase subunit sece | **SPN23F20300** |
|  | Putative protein-export membrane protein | Preprotein translocase subunit secg | **SPN23F08980** |
|  | Preprotein translocase secy subunit | Preprotein translocase subunit secy | **SPN23F02190** |
|  | Putative membrane protein | Preprotein translocase subunit yajc | **SPN23F03000** |
|  | Putative exported protein | Preprotein translocase subunit yajc | **SPN23F20500** |
|  | Membrane protein oxaa 1 precursor | Yidc/Oxa1 family membrane protein insertase | **SPN23F20630** |
|  | Putative preprotein seca subunit | Preprotein translocase subunit seca | **SPN23F17010** |
|  | Putative exported protein | Beta-lactamase class c | **SPN23F00100** |
|  | Putative extracellular oligopeptide-binding protein | Oligopeptide transport system substrate-binding protein | **SPN23F03390** |
|  | Riboflavin biosynthesis protein | Diaminohydroxyphosphoribosylaminopyrimidine deaminase / 5-amino-6-(5-phosphoribosyl amino)uracil reductase | **SPN23F01750** |
|  | Phospho-2-dehydro-3-deoxyheptonate aldolase, tyr-sensitive | 3-deoxy-7-phosphoheptulonate synthase | **SPN23F16990** |
|  | Response regulator protein blpr | Two-component system, lyttr family, response regulator agra | **SPN23F04780** |
|  | Pneumolysin (thiol-activated cytolysin) | Thiol-activated cytolysin | **SPN23F19470** |
|  | Competence-stimulating peptide type 2 precursor (csp 2) | Competence-stimulating peptide | **SPN23F22700** |
|  | Bacteriocin transport accessory protein | Competence factor transport accessory protein comb | **SPN23F00600** |
|  | Putative sensor histidine kinase | Two-component system, lyttr family, sensor histidine kinase comd | **SPN23F22690** |
|  | Sensor histidine kinase | Two-component system, ompr family, sensor histidine kinase ciah | **SPN23F07280** |
|  | Response regulator protein | Two-component system, ompr family, response regulator ciar | **SPN23F07270** |
|  | Putative response regulator protein | Two-component system, lyttr family, response regulator come | **SPN23F22680** |
|  | Putative membrane protein blpl | Immunity protein | **SPN23F04910** |
|  | Putative DNA-binding protein | HTH-type transcriptional regulator, regulator for comx | **SPN23F20100** |
|  | Putative competence-specific global transcription modulator | Competence protein comx | **SPN23F00150** |
|  | Peptide pheromone blpc (bacteriocin-like peptide) | Peptide pheromone blpc | **SPN23F04791** |
|  | ABC transporter blpb | Membrane fusion protein, peptide pheromone/bacteriocin exporter | **SPN23F04800** |
|  | Sensor histidine kinase blph | Two-component system, lyttr family, sensor histidine kinase agrc | **SPN23F04790** |
|  | Putative DNA-binding protein | HTH-type transcriptional regulator, SHP2-responsive activator | **SPN23F21530** |
|  | Putative regulatory protein | Hth-type transcriptional regulator, shp3-responsive repressor | **SPN23F09790** |
|  | Putative extracellular oligopeptide-binding protein | Peptide/nickel transport system substrate-binding protein | **SPN23F07780** |
|  | Tagatose 1,6-diphosphate aldolase 2 | Tagatose 1,6-diphosphate aldolase | **SPN23F10890** |
|  | S-ribosylhomocysteinase | S-ribosylhomocysteine lyase | **SPN23F03130** |
|  | Anthranilate synthase component I | Anthranilate synthase component I | **SPN23F18370** |
|  | Chromosomal replication initiator protein | Chromosomal replication initiator protein | **SPN23F00010** |
|  | Response regulator protein | Two-component system, ompr family, response regulator saer | **SPN23F00950** |
|  | ABC transporter, permease protein | Bacitracin transport system permease protein | **SPN23F08340** |
|  | Sensor histidine kinase protein | Two-component system, ompr family, sensor histidine kinase bras/bces | **SPN23F16340** |
|  | Response regulator protein | Two-component system, ompr family, response regulator protein brar/bcer | **SPN23F16350** |
|  | Putative activated D-alanine transport protein | Membrane protein involved in D-alanine export | **SPN23F22080** |
|  | D-alanyl carrier protein | D-alanine--poly(phosphoribitol) ligase subunit 2 | **SPN23F22070** |
|  | Putative D-alanyl-lipoteichoic acid biosynthesis protein | D-alanine transfer protein | **SPN23F22060** |
|  | Putative competence-specific global transcription modulator | Competence protein comx | **SPN23F00150** |
|  | Putative histidine sensor kinase | Two-component system, sensor histidine kinase yesm | **SPN23F01640** |
|  | Response regulator protein | Two-component system, response regulator yesn | **SPN23F01650** |
|  | Putative phage protein | Methyl-accepting chemotaxis protein | **SPN23F15730** |
|  | Sensor histidine kinase | Two-component system, narl family, sensor histidine kinase lias | **SPN23F03580** |
|  | Response regulator protein | Two-component system, narl family, response regulator liar | **SPN23F03590** |
|  | Histidine sensor kinase protein | Two-component system, narl family, sensor histidine kinase desk | **SPN23F20220** |
|  | Response regulator protein | Two-component system, narl family, response regulator desr | **SPN23F20210** |
|  | Alkaline phosphatase synthesis transcriptional regulatory protein | Two-component system, ompr family, alkaline phosphatase synthesis response regulator phop | **SPN23F21070** |
|  | Putative phosphate ABC transporter, extracellular phosphate-binding lipoprotein | Phosphate transport system substrate-binding protein | **SPN23F13650** |
|  | Sensor histidine kinase | Two-component system, ompr family, sensor histidine kinase vick | **SPN23F11220** |
|  | Response regulator protein | Two-component system, ompr family, response regulator vicr | **SPN23F11230** |
|  | Alanine racemase | Alanine racemase | **SPN23F16970** |
|  | Putative UDP-N-acetyl glucosamine-N-acetyl muramyl pyrophosphoryl-undecaprenol N-acetyl glucosamine transferase | UDP-N-acetylglucosamine-N-acetyl muramyl -(pentapeptide) pyrophosphoryl-undecaprenol N-acetylglucosamine transferase | **SPN23F06230** |
|  | Carbamate kinase | Carbamate kinase | **SPN23F21820** |
|  | Serine acetyltransferase | Serine o-acetyltransferase | **SPN23F05320** |
|  | Family carbohydrate kinase | 2-dehydro-3-deoxygluconokinase | **SPN23F02920** |
|  | Conserved hypothetical protein | 6-phosphogluconolactonase | **SPN23F14700** |
|  | Putative acylphosphatase | Acylphosphatase | **SPN23F19950** |
|  | Isopentenyl-diphosphate delta-isomerase | Isopentenyl-diphosphate delta-isomerase | **SPN23F03560** |
|  | Dihydroxy-acid dehydratase | Dihydroxy-acid dehydratase | **SPN23F21580** |
|  | Chorismate mutase type II protein | Chorismate mutase | **SPN23F11900** |
|  | NADP-specific glutamate dehydrogenase | Glutamate dehydrogenase (NADP+) | **SPN23F08900** |
|  | Putative coenzyme | Thiaminase (transcriptional activator) | **SPN23F06400** |
